# Visual reproduction test in normal elderly: Influence of schooling and visual task complexity

**DOI:** 10.1590/S1980-57642012DN06020005

**Published:** 2012

**Authors:** Paulo Roberto de Brito-Marques, José Eulálio Cabral-Filho, Rafael Moura Miranda

**Affiliations:** 1 Behavioral Neurology Unit, Department of Neurology, Faculty of Medical Sciences, University of Pernambuco, Recife PE, Brazil.; 2 Instituto Materno Infantil de Pernambuco (IMIP), Recife PE, Brazil.

**Keywords:** visual reproduction, visual memory, schooling

## Abstract

Tests of visual reproduction are used to assess visual memory. However, when the test is based on geometrical elements results could be influenced by schooling.

**Objective:**

To evaluate the influence of different schooling levels on performance of a visual reproduction task.

**Methods:**

A sample of 253 individuals (66 male and 187 female), aged 60 to 92 years were evaluated on a visual reproduction task comprising three geometric pictures of increasing complexity. Each individual was shown a picture for 8 to 10 seconds and a drawing of it was then immediately elicited. Four groups were defined according to the following schooling levels: illiterate, 1 to 4 years, 5 to 8 years and over 8 years. Individual performance was measured by summing the items correctly reproduced for the three pictures.

**Results:**

A significant difference for age was found between the illiterate and other three schooling groups. The reproduction of picture one was better reproduced than pictures 2 and 3 for all schooling levels (p<0.001). Pictures 2 and 3 did not differ among the schooling levels. Picture reproduction among the schooling levels showed that the group with over 8 years of schooling performed better on pictures 1 and 2 (p<0.001) but not on picture 3.

**Conclusion:**

Individuals aged 60 years or older, with 8 years' schooling or less, showed a reduced capacity to reproduce geometric pictures of a high degree of complexity. Clinical evaluations that use geometrical tests could be misinterpreted when not controlled for schooling level.

## Abstract (pt)

Teste de reprodução visual é usado para avaliar a memória visual, baseado em elementos geométricos e o resultado deve ser influenciado pela escolaridade.

**Objetivo:**

Avaliar os diferentes níveis de escolaridade durante a reprodução de tarefas visuais.

**Métodos:**

Uma amostra de 253 indivíduos (66 masculinos e 187 femininos), com idade entre 60 e 92 anos, foi avaliada em uma tarefa de reprodução visual composta por três desenhos geométricos de crescente complexidade. Cada indivíduo foi exposto a cada desenho geométrico de uma vez, e esse desenho foi reproduzido após 10 segundos de observação. Quatro grupos foram designados de acordo com o seguinte nível de escolaridade: analfabeto, 1 a 4 anos, 5 a 8 anos, e acima de 8 anos de escolaridade. A performance dos indivíduos foi medida pelos itens corretamentes reproduzidos.

**Resultados:**

Uma significante diferença da idade foi encontrada entre pacientes analfabetos e os outros três grupos de escolaridades diferentes. O desenho 1 foi reproduzido melhor do que os desenhos 2 e 3 em todos os níveis de escolaridade (p<0,001). Os desenhos 2 e 3 não diferiram em nenhum nível de escolaridade. A reprodução de um desenho entre níveis de escolaridade mostrou que o grupo acima de 8 anos de escolaridade realizou melhor nos desenhos 1 e 2 (p<0,001), mas não foi no desenho 3.

**Conclusão:**

Indivíduos com idade de 60 anos ou mais com até 8 anos de escolaridade mostrou capacidade diminuída na reprodução de desenhos geométricos de alto grau de complexidade. A avaliação clínica usando testes geométricos poderia ser mal interpretada quando a escolaridade não estiver de acordo com o grau de complexidade do teste.

## INTRODUCTION

According to the definition of Lezak,^1^ visual memory is the capacity to retain information and utilize it for adaptive purposes. Efficient visual memory requires the intact functioning of many specific brain regions. The use of the same term – memory – to denote some very different mental activities can lead to confusion. Normal elderly people have been diagnosed with visuospatial dysfunctions under the umbrella of visual memory impairment. During a visual memory test, the examiner should be able to estimate the relative contributions of perception and constructional skills as well as visual memory to the final result. Visual stimuli should not be so complex that only an exceptional person would be able to perceive and retain them with only one exposure of a few seconds. Recent studies of visual perception are bringing us closer to an understanding of what is remembered – and what is forgotten – during the recalling of a scene.

Memory can be defined as the recording, retention, and retrieval of knowledge. It accounts for all knowledge gained from experience – facts that are known, events that are remembered, and skills that are applied. Temporal properties also distinguish one form of memory from another. At least two stages are recognized in the construction of memories: short-term memory and long-term memory. However, several distinctions should be made among aspects of memory that are useful from a clinical perspective and from a neuroscientific perspective.^3-6^ Immediate memory refers to the recalling of information without delay, either immediately after presentation or uninterrupted rehearsal. Immediate memory features the ability to store information during a given situation while other information is not used.^4-7^ Some authors believe that immediate memory comprises a memory system which can transfer information like a limited capacity retrieval system.^4-6,8^

The topography underlying executive functions is located in the frontal lobes, particularly the prefrontal lobes. Dysexecutive syndrome encompasses five types of disorder:

[1] Deficits in initiation, cessation, and control of action;
[2] Impairments in abstract and conceptual thinking;
[3] Deficits in cognitive estimation;
[4] Lack of cognitive flexibility and deficits in response to new information; and
[5] Deficits in goal-directed behaviors.^9,10^

Executive functions consist of those capacities that enable a person to engage successfully in dependent, purposeful, self-serving behavior. Provided the executive functions are intact, a person can sustain considerable cognitive loss and still continue to be independent, constructively self-serving, and productive. When executive functions are impaired, the individual may no longer be capable of carrying out satisfactory self-care, performing remunerative or useful work independently, or able to maintain normal social relationships regardless of how well-preserved their cognitive capacities are – or how high the person scores on tests of skills, knowledge, and abilities.^11^

Visual memory tests can be impaired among aged groups as a result of other factors besides visual memory. According to Lezak,^1^ such tests often call for a visuomotor response, typically drawing. This can complicate the interpretation of performance deficits since failure may arise from constructional disability, impairment of visual or spatial memory, or from an interaction among these and other factors.

Many types of problems drawing printed geometric pictures have been described under the name of constructive apraxia. Indeed, the term has been used to designate both drawing disability and defective execution of many other kinds of constructional tasks.^12^ Tests based on geometric pictures require not only preserved attention and STM, but also perception and visuoconstructive skills. Therefore, three abilities are required to allow adequate responses to questions posed in this kind of test: memory, praxia and perception.

Although visual memory tests have been used for individuals of different schooling levels, it is important to investigate visual memory by using tests of reproduction of geometrical pictures according to level of schooling. Some of these tests require the ability to draw geometric figures. On the other hand, the reconstruction of a geometric figure should be easier for individuals with greater schooling since this ability is part of education *curricula*. The relationship between visual reproduction tests and schooling level could be important because of the risk of misinterpretation of results, having serious repercussions on diagnostic decisions. False negative or false positive results could arise if the relationship between educational level and test performance is not taken into account. However, to our knowledge, few studies investigating this issue in normal elderly are available in the literature. Therefore, an investigation on this topic is opportune. The aim of the present study was to test the hypothesis that individuals with greater schooling perform better than individuals with lower schooling on visual memory tests.

## METHODS

**Figure 1:**
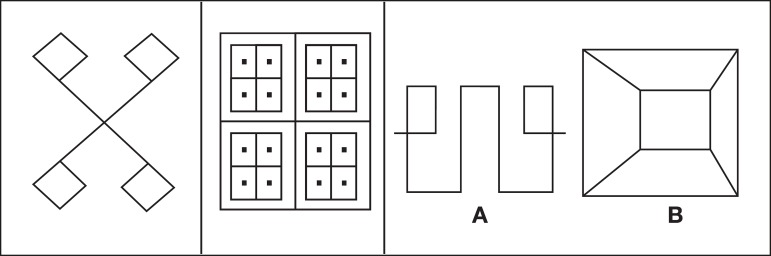

A randomized cross-sectional study was conducted enrolling 253 individuals of both genders (66 male and 187 female), 60 to 92 years old, belonging to low and middle socio-economic classes, from the city of Olinda, Pernambuco, Brazil. Visual memory was assessed using a visual reproduction test according to Wechsler Memory Scale-Revised.^13^ The procedure was performed as an immediate recall test. The short test consisted of observing and drawing three printed geometric pictures of increasing complexity. Complexity was defined by a higher number of graphical components. This sub-test assesses the skills of memorization and reproduction of visual stimuli (Figure 1).

The test contains 3 pictures presented on separate cards. The first picture is scored from zero to 3 points: 1 point given for two lines crossing with four flags; 1 point if flags have the correct position facing each other at the top and bottom; 1 point if correct proportions are observed in relation to the central angle (which must be between 60º and 120º) and to dimensions of the 4 sides of each flag (flags should be in the form of a square).

The second picture is scored from zero to 5 points: 1 point for the presence of the bigger outer square, properly divided by perpendicular lines crossing through the middle; 1 point for the presence of 4 medium-sized squares within the large square; 1 point for division of the medium squares by perpendicular lines crossing through the middle; 1 point for the presence of a central dot in each of the 16 small squares; and 1 point for observing the correct proportions of size among the squares.

The third picture task is divided into to two distinct parts (a and b) and scored from zero to 7 points: Part a – 1 point if the central rectangle is open in the correct places with good lines extending to the side loops; 1 point for the existence of two more or less correct loops, forming a right angle with the lines derived from the rectangle; 1 point if the two symmetrical loops are placed within the inner part of the drawing; 1 point if the proportions are respected where the height of the inner rectangle should be close to that of the arms supporting the loops. Part b – 1 point for reproduction of the large rectangle with small inner rectangle according to their geometrical forms; 1 point is given if the top of the inner rectangle is joined to the top of the outer rectangle by straight lines; 1 point for reproduction of the correct parallelism between the two rectangles. The maximum score for the 3 picture test is 15 points.

Before being shown each picture, the subject was informed about the test procedure. The picture was then presented for eight to ten seconds and immediately removed from view. Soon after this visual presentation, the individual was required to reproduce the picture manually on a white sheet of paper. Following each picture exposure, the individual drew what they remembered of the picture. For the test, each picture was showed separately starting with the image of lowest complexity. Each picture was scored by summing the items correctly reproduced, adding subtotals to give a final score for the individual, considering the maximal value of 100% in each picture.

A clinical interview was performed with each subject by one of the authors (PRBM) in order to investigate neurological and psychiatric diseases. To verify praxia ability, all individuals reproduced a copy of a circle and a square before testing. Since mentally disabled people have difficulty performing some tasks of daily living, participants were queried about their normal daily routine. Individuals unable to get around, tell the time on a clock, handle cash money, or use a tin opener (intermanual conflict) were excluded. People with low visual or auditory acuity, motor or rheumatic disturbance, chronic alcoholism, cardiovascular disease, recent head trauma (last 12 months) or a lack of motivation, were also excluded.

To verify the influence of schooling on visual reproduction, four groups were formed according to schooling level: Illiterate Group (n=28), with mean age of 73.7 (SD=6.0) years, comprising individuals with no formal schooling; schooling Group 1-4 (n=119), with mean age of 70.2 (SD=7.2) years – individuals with 1 to 4 years of formal instruction; Group 5-8 (n=85), with mean age of 67.6 (SD=5.7) years – individuals with 5 to 8 years of formal education; and a Group over 8 years (n=21), with a mean age of 66.4 (SD=6.5) years – individuals with over 8 years of formal education. This study was approved by the Research Ethics Committee of the University Oswaldo Cruz Hospital in the city of Recife – Brazil.

**Statistical analysis.** Prior to statistical analysis, the variance homogeneity and normality of the data were verified by Levine and Kolmogorov-Smirnov tests, respectively. When these conditions were not met and groups were dependent, Friedman's analysis followed by Dunn's test for multiple comparisons of median between each pair of two groups, was applied. When the data satisfied the criteria of normality Student's "t" test was used.

The alpha error for rejection of the null hypothesis was 0.05.

## RESULTS

Comparison of the difference in age among the schooling groups (Table 1), revealed that illiterate individuals had a higher mean age than those from the 1 to 4 years (p<0.05), 5 to 8 years (p<0.001), and over 8 years (p<0.001) schooling groups. However, there was not statistical difference among the literate groups.

**Table 1:** Age comparisons by schooling level.

	Illiterate (n=28)	1-4 years (n=119)	5-8 years (n=85)	>8 years (n=21)
X±SD	73.7±6.0	70.2±7.2	67.6±5.7	66.4±6.5

Comparisons among levels: ANOVA. Multiple comparisons of each pair of levels. Tukey's test. Illiterate X (1-4 y), p<0.05; Illiterate X (5-8 y), p<0.001; Illiterate X (>8 y), p=0.001.

**Figure 2:**
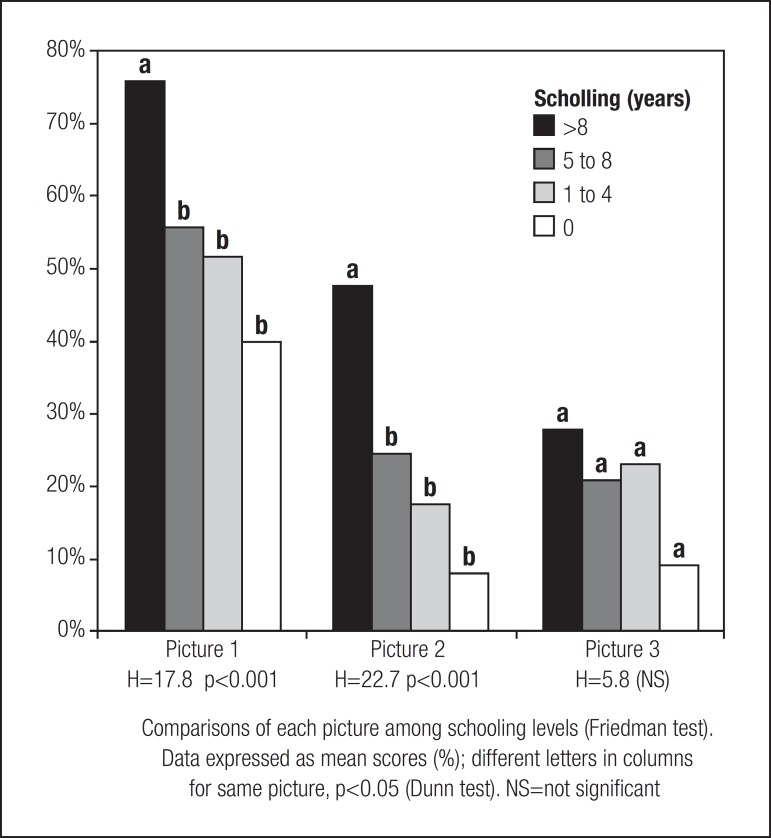
Visual reproduction by individuals, aged 60-92 years, with different schooling levels according to picture complexity.

Concerning the picture reproduction by the four schooling levels (Figure 2), results verified that subjects with a schooling level of over 8 years performed better for pictures 1 and 2 (p<0.001) but not for picture 3, compared with the other schooling levels. There were no statistical differences among the three other schooling levels for any of the pictures.

**Figure 3:**
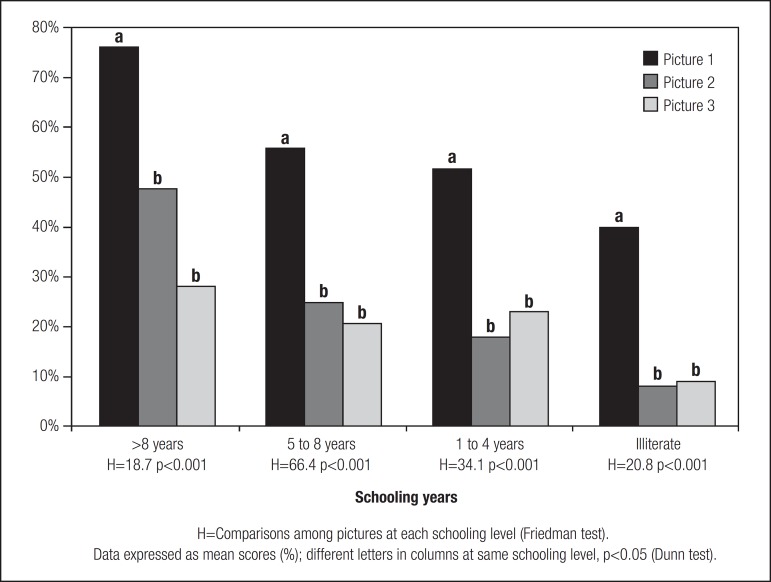
Visual reproduction of geometrical pictures of increasing complexity by individuals aged 60-92 years, according to schooling level.

Upon examining the three pictures at each schooling level (Figure 3), it was observed that only Picture 1 differed compared to the others.

## DISCUSSION

Several studies have shown an association of the execution or reproduction of mental and memory tasks with schooling level.^11-16^ The process of visual reproduction of a picture after a time interval, such as that used in the present study, is dependent on memory, especially short-term memory.^16^ Moreover, the relationship between schooling and visual acquisition may influence the reliability of reproduction.^17-19^ Our results show that individuals with over 8 years of schooling had superior performance to the other groups on visual reproduction of Pictures one and two (Figure 2). This is an important finding because it could indicate that schooling level influences performance on the test. On the other hand, the illiterate group had an overall lower performance but was significantly older than the over 8 years schooling group. In this case, the influence of age on the result observed cannot be ruled out, thus precluding confirmation of an effect of schooling on the performance of these individuals. Nevertheless, the fact that pictures one and two yielded a difference in scores between the highest schooling group (>8 years) and the other schooling levels (Figure 2) allows the deduction that, for the reproduction of the pictures one and two, individuals perceive the basic principle of their construction more easily than that of picture three. Indeed, the first two pictures each present only one focus of attention, while picture three presents two foci. It is probable that to draw more reliably a geometric picture from the observation of another similar picture, some acquired hand skill or innate intellectual ability could be relevant, a skill which could be acquired during school training. In addition, it has been demonstrated that the memory of a scene can be influenced by the meaning of that scene.^2^

Considering the variations in complexity of the pictures, it was verified that the best performance was observed for Picture one for all schooling levels, while the visual reproduction of Pictures two and three was equivalent for all schooling levels, despite being lower than that of Picture one (Figure 3). The superior reproduction of Picture one for all schooling levels could suggest that other factors other than schooling influence visual reproduction. The most important factors for the reproduction of pictures displayed in this test are drawing skill, attention, holistic perception of the picture, and visual memory.^1^

The lower familiarity of the less schooled individuals regarding handling of school implements (pencil, pen, paper, etc.) could have induced an apparent constructive apraxia or incapacity to draw, especially in relation to complex pictures.

In healthy individuals, visual memory is a dynamic cognitive component dependent on a number of cognitive and functional cortical components that contribute to the establishment of information for the formation of immediate memory and short-term memory. Visual memory may involve several components such as language, praxia, supported by attention, perception, and motivation. In individuals with neurodegenerative diseases, as well as Pick's disease, Lewy body disease or corticobasal degeneration that lead to loss of visual memory, changes are evident on the tests in various ways. It is believed the functional structure of the cerebral cortex can change the extent to which the degenerative disease progresses. Thus, it is possible to change the visual memory task period according to the time the test was performed in the disease course.^20-22^ According to Lezak,^1^ since the visual memory impairments can take on a variety of forms in different age groups, no single assessment technique demonstrates the problem for all individuals. Moreover, the quality of an individual's response compared to other neuropsychological measures should enable the examiner to estimate the relative contributions of perception, constructional skill, and memory to the final result.

Although the four schooling groups did not differ significantly on the reproduction of the picture of highest complexity, this observation warrants attention because the performance of people aged 60 or older with low schooling (less than 8 years of schooling), such as those studied, could be misinterpreted when submitted to a complex visual reproduction test. Therefore, elderly people with low schooling level should be recommended – when assessed during clinical examination – to reproduce only the simplest picture. This is an important concern in preventing false-positive results from being produced, with negative ramifications for the individual evaluated if this is not taken in account.

We reported a preliminary approach concerning neuropsychological responses of elderly people with different levels of schooling for visual reproduction of geometric pictures. A more in-depth exploratory investigation of this problem which includes people with memory complaints should be performed.
